# *Epichloë* fungal endophyte interactions in perennial ryegrass (*Lolium perenne* L.) modified to accumulate foliar lipids for increased energy density

**DOI:** 10.1186/s12870-023-04635-8

**Published:** 2023-12-11

**Authors:** Kim A. Richardson, Anouck C. M. de Bonth, Zac Beechey-Gradwell, Suhas Kadam, Luke J. Cooney, Kelly A. Nelson, Ruth Cookson, Somrutai Winichayakul, Michele Reid, Philip Anderson, Tracey Crowther, Xiuying Zou, Dorothy Maher, Hong Xue, Richard W. Scott, Anne Allan, Richard D. Johnson, Stuart D. Card, Wade J. Mace, Nicholas J. Roberts, Gregory Bryan

**Affiliations:** 1 Resilient Agriculture, AgResearch Ltd, Palmerston North, 4442 New Zealand; 2 Division of Plant Sciences & Technology, University of Missouri, Columbia, 65201 MO USA; 3 Division of Plant Sciences & Technology, University of Missouri, Novelty, 63460 MO USA; 4 Present address: SGS North America, Crop Sciences, Brookings, SD 57006 USA

**Keywords:** *Lolium perenne*, *Epichloë*, Lipids, Metabolizable energy

## Abstract

**Background:**

Commercial cultivars of perennial ryegrass infected with selected *Epichloë* fungal endophytes are highly desirable in certain pastures as the resulting mutualistic association has the capacity to confer agronomic benefits (such as invertebrate pest deterrence) largely due to fungal produced secondary metabolites (e.g., alkaloids). In this study, we investigated T_2_ segregating populations derived from two independent transformation events expressing diacylglycerol acyltransferase (DGAT) and cysteine oleosin (CO) genes designed to increase foliar lipid and biomass accumulation. These populations were either infected with *Epichloë festucae* var*. lolii* strain AR1 or *Epichloë* sp. LpTG-3 strain AR37 to examine relationships between the introduced trait and the endophytic association. Here we report on experiments designed to investigate if expression of the DGAT + CO trait in foliar tissues of perennial ryegrass could negatively impact the grass-endophyte association and vice versa*.* Both endophyte and plant characters were measured under controlled environment and field conditions.

**Results:**

Expected relative increases in total fatty acids of 17–58% accrued as a result of DGAT + CO expression with no significant difference between the endophyte-infected and non-infected progeny. Hyphal growth in association with DGAT + CO expression appeared normal when compared to control plants in a growth chamber. There was no significant difference in mycelial biomass for both strains AR1 and AR37, however, *Epichloë*-derived alkaloid concentrations were significantly lower on some occasions in the DGAT + CO plants compared to the corresponding null-segregant progenies, although these remained within the reported range for bioactivity.

**Conclusions:**

These results suggest that the mutualistic association formed between perennial ryegrass and selected *Epichloë* strains does not influence expression of the host DGAT + CO technology, but that endophyte performance may be reduced under some circumstances. Further investigation will now be required to determine the preferred genetic backgrounds for introgression of the DGAT + CO trait in combination with selected endophyte strains, as grass host genetics is a major determinant to the success of the grass-endophyte association in this species.

**Supplementary Information:**

The online version contains supplementary material available at 10.1186/s12870-023-04635-8.

## Introduction

Perennial ryegrass (*Lolium perenne* L.) is primarily grown to support the nutritional requirements of livestock under grazing in temperate pastoral agricultural systems as a result of its rapid establishment, long growing season, high palatability, and digestibility for ruminant animals when compared to alternative perennial grass species [1]. Continuing improvements through plant breeding are essential to the enhancement of on-farm productivity for this species. Annual genetic gains ranging from 0.3 to 0.75% have been reported for traits such as dry matter yield and nutritive value through conventional breeding programmes, and this has resulted in the release of many commercial cultivars over the past four decades [2, 3]. However, it is also recognised that biotechnology holds the promise of providing rapid additional genetic gain in this species through the introduction of novel traits [4]. Biotechnology approaches through the expression of chimeric genes in ryegrass have the potential to address abiotic stressors such as drought or salinity [5, 6], virus resistance [7], improved nutritional composition through the accumulation of water soluble carbohydrates [8], altered fibre composition [9], and the accumulation of stable lipids in leaf tissues using DGAT + CO technology [10].

The expression of DGAT + CO was developed to increase the energy content of plants through an elevation in fatty acid (FA) concentration in foliar tissues and was first described in Arabidopsis [11]. Stable accumulation is achieved through the combined expression in green tissues of a diacylglycerol O-acytltransferase 1 (DGAT) gene which increases lipids in the form of triacylglycerides, and a cysteine oleosin (CO) gene which encapsulates the triacylglycerides to form oil bodies resistant to degradation in the cell. The DGAT + CO technology targets FA as they contain more joules per gram than carbohydrates or protein, and the supplementation of lipids in the ruminant diet reduces production of methane and improves the FA composition of meat and milk [12]. Subsequently, DGAT + CO expression was demonstrated in perennial ryegrass [10, 13, 14]. Relative increases of up to 75% in FA were observed along with a corresponding increase in photosynthesis, specific leaf area, and relative growth rates in perennial ryegrass modified with DGAT + CO when grown in a spaced pot arrangement [14]. Consistent with studies assessing the effects of increased fat in ruminant diets, a 10–15% decrease in methane as a proportion of total gas production was observed during in vitro digestion with rumen fluid [15].

Commercial cultivars of perennial ryegrass and tall fescue are often intentionally infected with selected strains of the *Epichloë sp.* mutualistic endophytic fungi, which are known to increase the agronomic competitiveness of the host grass [16, 17]. These selected *Epichloë* strains contribute to the productivity of pastoral agriculture in New Zealand, Australia and the USA [18] which is largely achieved through the *in planta* production of secondary metabolites, most notably alkaloids derived from these fungi [19]. Some *Epichloë* strains, including the common-toxic strain identified in New Zealand have the capacity to cause mammalian toxicities, including ryegrass staggers, heat stress in sheep and cattle, and lower livestock productivity (for review read [19]). However, a wide range of chemotypic diversity exits among and even within species of *Epichloë* and selected strains devoid of mammalian toxins or with less toxic alkaloid profiles can confer protection against biotic or abiotic stresses with minimal ill effects to livestock. Such strains are deployed in combination with elite pasture grass cultivars to enhance pasture performance. For instance, *Epichloë* strains expressing high levels of alkaloids such as peramine (e.g. AR1) or epoxyjanthitrems (e.g. AR37) in the absence of mammalian toxins, such as lolitrem B or ergovaline, are selected for use in pastures to provide broad spectrum insect resistance while avoiding livestock toxicoses [20, 21].

Asexual forms of *Epichloë* do not extend outside of the plant host, colonising only the aerial organs. They grow within the intracellular spaces of their host in a highly regulated manner and are strictly vertically transmitted to the seed via the gynecium during flowering [22]. Following seed germination, the fungus spreads systemically throughout the aerial portions of the plant forming a permanent mutualistic association [23]. However, vertical transmission frequency in some strains is heavily influenced by the grass host genetics, which generates an often-imperfect process resulting in endophyte-free progeny possessing a reduced agronomic fitness in the presence of certain insect pests [24, 25].

Agricultural forages developed from genetic modifications are subject to strict regulatory processes and require rigorous testing prior to deregulation to demonstrate human, animal, and environmental safety [26, 27]. These regulations are used to demonstrate that no unintentional toxicity or allergenicity has been introduced due to the modification. Typically, the nutritional composition of the modified crop is compared to a commercial non-modified cultivar of similar genetics, with a history of safe use, to demonstrate a substantial equivalence. For perennial ryegrass modified with DGAT + CO, this rigour will apply not only to a compositional assessment of the plant but also to the associated *Epichloë* strains to ensure that mycelial biomass and alkaloid production falls within the expected range for (non-modified) commercial cultivars.

The objective of this study was to investigate the impacts of FA accumulation in foliar tissues of perennial ryegrass resulting from DGAT + CO expression upon its mutualistic association with selected strains of *Epichloë* and vice versa in growth chamber and field environments. We examined hyphal growth and biomass, vertical transmission frequency, and secondary metabolite production of *E. festucae* var. *lolii* strain AR1 and *Epichloë* sp. LpTG-3 strain AR37 in T_2_ populations segregating for DGAT + CO and the corresponding null-segregant progeny. FA accumulation, relative growth rate and herbage yield of the perennial ryegrass host were also measured. To our knowledge this is the first instance where the symbiotic relationship between a forage grass host and its *Epichloë* endophyte has been characterised in the field following introduction of a host trait using recombinant technology. The results provide useful insights into the management of endophyte transmission during the genetic stabilisation of DGAT + CO and breeding phase of this important forage trait.

## Results

### Leaf fatty acid profiles

**Fig. 1:**
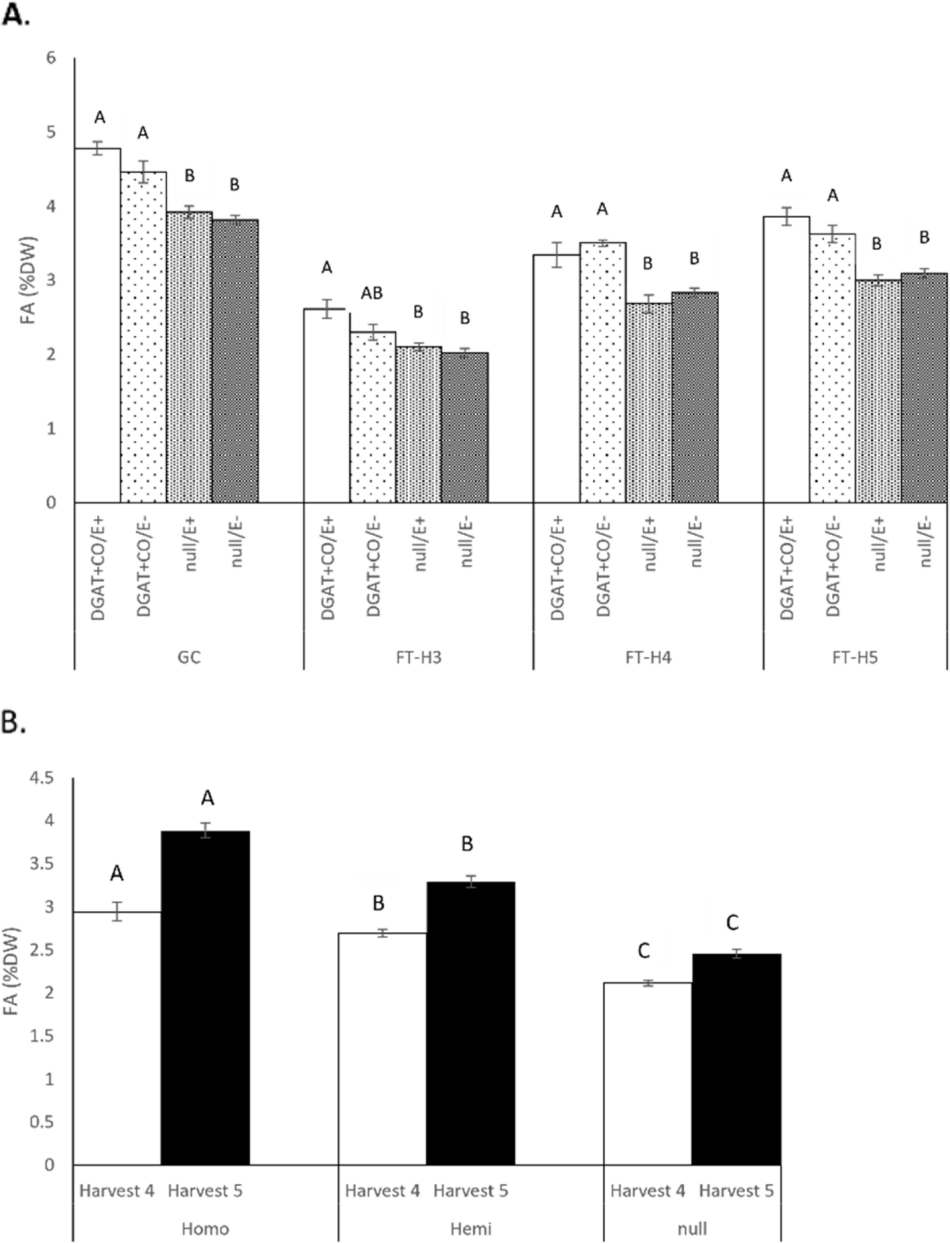
**A**. Mean total fatty acid (FA) concentration for AR1- infected DGAT + CO T_2_ and null plants grown in a growth chamber (GC, *n* = 6) and in single field mini-swards in 2019 (FT, harvests (H) 3 to 5, *n* = 9) in the presence (E +) or absence (E-) of endophyte strain AR1. **B**. Mean total fatty acid by zygosity for AR37-infected DGAT + CO T_2_ plants grown in replicated field miniswards in 2021 (FT, H 4 and 5, *n* = 7). Bars represent means ± SE. Letters A and B indicate a significant difference at *P* < 0.05, within each harvest of the trial

Plants segregating for DGAT + CO displayed significantly (*P* < 0.05) higher leaf FA concentrations than the relevant null plants, depending on harvest date (Fig. 1). This ranged from 17% for DGAT + CO/endophyte-free (E-) to 25% for DGAT + CO/endophyte-infected (E +) compared to the relevant null/E- in growth chamber grown plants, and between 13 and 29% for DGAT + CO/E- and DGAT + CO/E + , respectively, for plants in the AR1-infected 2019 field experiment (Fig. 1A). The total FA of plants grown in a growth chamber was 4.5 and 4.8% of leaf dry weight (DW) in DGAT + CO for E- or E + plants, respectively, while the null plants ranged from 3.8 to 3.9% of leaf DW (Table 1). Additionally, the FA composition of the DGAT + CO plants was altered, with significant (*P* < 0.05) increases in the ratios of long-chain FA (C18:1 and C18:2) and a decrease in the ratio of C18:3 (Table 1). The presence of *Epichloë* did not influence FA accumulation in these plants (*P* > 0.05). Similarly, for plants grown in field miniswards, the total FA represented 2.3 to 3.9% of leaf DW in DGAT + CO plants while the null plants ranged from 2.0 to 3.1% of leaf DW depending upon harvest and endophyte status (Fig. 1A).

**Table 1:** Fatty acid profile (% total FA) and total fatty acids (% Leaf DW) in DGAT + CO and null plants grown in a growth chamber (*n* = 6) where all plants are either infected or non-infected with AR1 fungal endophyte strain. Means (± SE) with letters A and B indicating a significant difference within the FA class at *P* < 0.05)

	C16:0	C16:1	C18:0	C18:1	C18:2	C18:3	Total FA (%DW)
DGAT + CO/E +	12.72 (0.08)	2.01 (0.04)	1.08 (0.03)	5.02^A^ (0.40)	20.91^A^ (0.40)	58.26^A^ (0.32)	4.78^A^ (0.09)
DGAT + CO/E-	12.72 (0.08)	2.01 (0.04)	1.08 (0.03)	5.02^A^ (0.40)	20.91^A^ (0.40)	58.26^A^ (0.32)	4.78^A^ (0.09)
null/E +	13.36 (0.15)	2.23 (0.08)	1.59 (0.26)	1.72^B^ (0.23)	11.79^B^ (0.48)	69.31^B^ (0.66)	3.92^B^ (0.09)
null/E-	13.42 (0.22)	2.29 (0.08)	1.48 (0.21)	1.74^B^ (0.39)	11.66^B^ (0.51)	69.40^B^ (1.18)	3.82^B^ (0.06)

By comparison the transformation event used in the AR37-infected 2021 field experiment displayed significant increases in FA which were incremental depending on zygosity of the DGAT + CO trait and varied for each harvest. Significant (*P* < 0.05) increases of 27 and 39% FA were observed in hemizygous and homozygous plants respectively compared to the null-segregants for Harvest 4, and 34 and 58% for Harvest 5 (Fig. 1B). In accordance with the results observed for growth chamber grown plants; the FA composition of all field grown plants, regardless of endophyte strain (with the exception of Harvest 4 for AR37) had significant (*P* < 0.05) increases in long-chain FA C18:1 and C18:2, and a decrease in the ratio of C18:3, while the endophyte status did not impact FA accumulation (*P* > 0.05) (Supplementary Table S1).

### Herbage growth rate

**Fig. 2:**
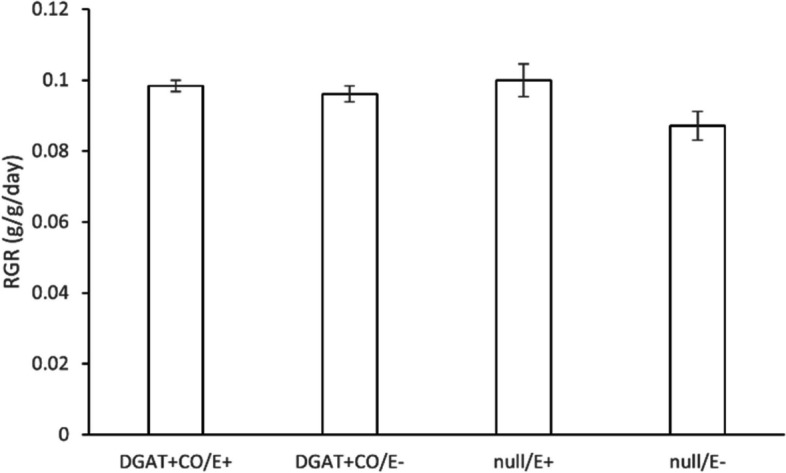
Relative growth rate for a segregating population of T_2_ DGAT + CO and null plants grown in a growth chamber. Plants were either infected (E +) or non-infected (E-) with AR1 fungal endophyte strain. Bars are means ± SE, *n* = 6

Biomass accumulation was measured in AR1-infected and E- progeny, of a DGAT + CO event, grown in a controlled growth chamber environment in order to validate previous results where increases in relative growth rate (RGR) were demonstrated for DGAT + CO events (both in the T_0_ primary transgenic and T_2_ generation) [13, 14, 28]. No significant differences (*P* < 0.05) in RGR between DGAT + CO or null-segregants were observed for E + or E- plants when grown in a dense sward arrangement in a growth chamber (Fig. 2). Similarly, no significant difference (*P* < 0.05) in herbage yield was detected in the field between DGAT + CO or null-segregant swards regardless of endophyte status (Supplementary Figure S1).

### Confocal microscopy of hyphal growth

**Fig. 3:**
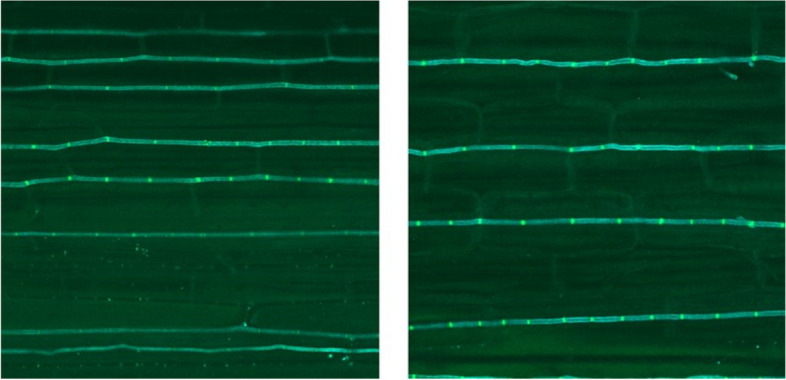
Confocal micrograph showing hyphae of *E. festucae* var*. lolli* strain AR1 in a leaf sheath of DGAT + CO (left) and null (right) ryegrass at 60-fold magnification

Null-segregant and DGAT + CO T_2_ progeny collected from E + parents were also used to visualise hyphal growth in fluorescently stained leaf sections *in planta* under confocal microscopy at tenfold and 60-fold magnification. Six genotypes each of DGAT + CO and the corresponding null-segregant progeny were sectioned. All microscopy observations recorded viable and abundant hyphae which displayed normal growth patterns for both DGAT + CO and null controls at a similar developmental stage when viewed in a longitudinal section (Fig. 3). No hyphae were detected in E- controls (data not shown).

### Systemic colonisation and vertical transmission of endophyte

The process of vertical transmission from the seed into the seedling and then systemic colonisation of the selected endophyte strains into the developing daughter tillers within plants was examined within DGAT + CO to determine if a successful ryegrass-*Epichloë* association could be established for the colonisation of reproductive structures. At least 10 randomly selected tillers/plant from a total of 24 plants (originating from the AR1-infected T_2_ populations) were examined for the presence of viable *Epichloë*. Overall, 240 tillers (120 DGAT + CO, 120 null-segregant) were identified as positive for the presence of *E. festucae*. var *lolii* AR1, indicating no differences in systemic colonisation between the DGAT + CO and null segregant seedlings. For vertical transmission, the transfer frequency of viable endophyte from the DGAT + CO and Ceres One50 parents to seedling progeny are shown in Table 2. In general, the frequency of AR1 transmission in each of the four crosses was within the range obtained for the control pairwise cross between a non-transformed Alto genotype and Ceres One50 parent.

**Table 2:** Vertical seed transmission of AR1 into T_3_ progeny from four DGAT + CO/E + T_2_ parents crossed pairwise to individual genotypes of Ceres One50/AR1

	% Endophyte infection (infected/total)	
	DGAT + CO T_2_ parent	Ceres One50 parent
Alto/AR1(DGAT + CO x Bronsyn)_1_ × One50_1_/AR1	100 (14/14)	94 (17/18)
Alto/AR1(DGAT + CO x Bronsyn)_2_ × One50_2_/AR1	94 (16/17)	81 (13/16)
Alto/AR1(DGAT + CO x Bronsyn)_3_ × One50_3_/AR1	92 (12/13)	100 (16/16)
Alto/AR1(DGAT + CO x Bronsyn)_4_ × One50_4_/AR1	100 (17/17)	100 (13/13)
Alto/AR1 x One50_5_/AR1	94 (16/17)	94 (17/18)

### Quantification of endophyte biomass and selected alkaloids

**Fig. 4:**
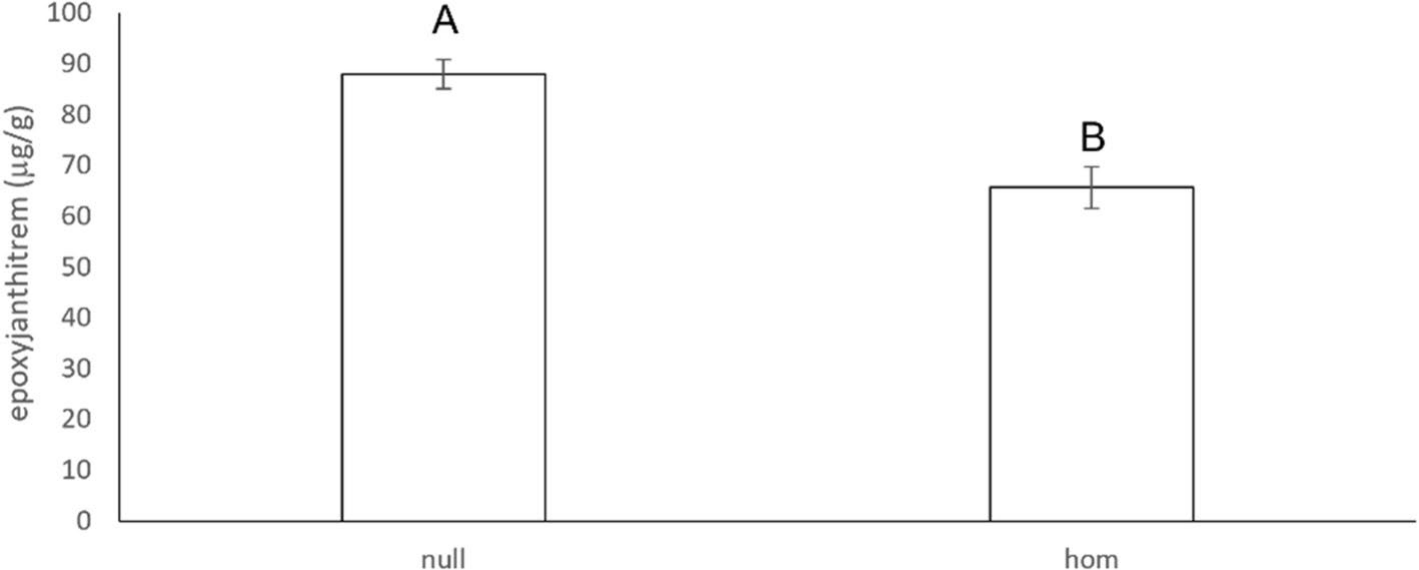
Mean values for total epoxyjanthitrem concentration (± SE) from AR37 infected T_3_ seed of either homozygous (hom) DGAT + CO or the corresponding null population. *n* = 29 seed samples from controlled crosses. Letters indicate significant difference at *P* < 0.05

Quantitative analysis for *Epichloë* mycelial biomass by competitive ELISA and relevant alkaloids using LC–MS/MS are shown in Table 3 (full analysis shown in Supplementary Table S2). In general, the reduction in *Epichloë* mycelial biomass observed in DGAT + CO plants was not significantly different (*P* < 0.05) compared to the null-segregants for AR1 or AR37, grown in the growth chamber or field. However, peramine concentrations in AR1-infected plants were significantly (*P* < 0.05) reduced by approximately 30% in DGAT + CO leaf tissue harvested from both growth chamber grown plants and in Harvest 5 of the field trial (Table 3A). For AR37-infected plants, a 40% and 31% reduction in epoxyjanthitrems was observed for the homozygous DGAT + CO in Harvest 4 when compared to the null-segregants and hemizygous DGAT + CO, respectively (Table 3B). Similarly, a 25% reduction in total epoxyjanthitrem concentration was observed in seed from the T_3_ generation for homozygous DGAT + CO when compared to a genetically identical null population (Fig. 4).

**Table 3:** **A**. Mean values for AR1 mycelial mass and peramine concentration (± SE), in T_2_ progeny segregating for DGAT + CO grown either in a growth chamber or in the field. **B**. Mean values for AR37 mycelial mass and total epoxyjanthitrem concentration (± SE), by zygosity of the DGAT + CO trait in T_2_ segregating progeny grown in the field. Bars represent means ± SE. Letters A and B indicate a significant difference at *P* < 0.05 comparing DGAT + CO hemizygous, homozygous, or null progeny

A			
AR1			
Mycelial mass (µg/mL)		DGAT + CO	Null
Growth Chamber	Pseudostem	2.10 (0.36)	2.07 (0.18)
	Leaf	1.53 (0.26)	1.75 (0.25)
Field	Harvest 3	1.14 (0.12)	1.25 (0.16)
	Harvest 4	1.59 (0.23)	1.76 (0.20)
	Harvest 5	1.00 (0.20)^B^	1.13 (0.09)^A^
B			
AR37 (Field)			
Mycelial mass (µg/mL)	Homozygous (DGAT + CO)	Hemizygous (DGAT + CO)	Null
Harvest 4	2.77 (0.12)	3.02 (0.20)	3.19 (0.25)
Harvest 5	2.44 (0.15)	2.90 (0.34)	2.83 (0.21)
Total epoxyjanthitrem (µg/g)			
Harvest 4	22.70 (1.83)^B^	33.04 (1.55)^A^	37.54 (3.03)^A^
Harvest 5	19.47 (2.24)	25.26 (2.48)	26.64 (1.88)

## Discussion

The expression of DGAT + CO in perennial ryegrass has been well documented with a FA increase of 14–34% in foliar tissues which translates into a 0.2–0.5 kJ/gDW increase in herbage gross energy (GE) measured in the field [10, 13, 14, 28]. We speculate that the DGAT + CO events used in these experiments with an FA enhancement of up to 58% could translate to almost 1 kJ of additional GE to meet the goal of developing DGAT + CO for the improvement of animal nutrition. This would be feasible, especially where DGAT + CO has been stabilized in a homozygous form within the population. Moreover, an assessment of the DGAT + CO trait for its potential to reduce methane emissions, using a proxy in vitro assay with a 10–15% decrease in methane production during in vitro digestion with rumen fluid, has been described for some events which supports the additional potential benefit of the trait in ruminant nutrition [15]. The advantages conferred by DGAT + CO has the potential to play an important role in boosting the genetic gain of perennial ryegrass for energy availability and mitigation of methane emissions from ruminants.

### Transmission of endophyte

Seed production to introduce AR1 and AR37 into a DGAT + CO genetic background by controlled-pair crosses using infected non-modified maternal parent plants was necessitated by the self-incompatible, outcrossing nature of perennial ryegrass. The AR1-infected population was developed by harvesting seed from both parents to allow a direct comparison of the endophyte in a DGAT + CO background compared to the null-segregant, and of DGAT + CO with E + and E- endophyte infection. Progeny from both parent plants inherited DGAT + CO at approximately 1:1 with 100% endophyte infection observed in seed collected from the E + parent based upon analysis by PCR, immunoblotting for DGAT1 and the presence of the endophyte. For AR37, the population was generated by crossing T_1_ genotypes to produce progeny segregating 3:1 for DGAT + CO and 100% transmission of the endophyte, which was determined using the same analyses used for the AR1 population.

### Plant traits

The significant increases in foliar FA content observed in DGAT + CO progeny when compared to null progeny were consistent with those reported previously [11, 14, 28]. DGAT + CO/AR1 progeny exhibited a 22–25% increase in leaf FA content compared to the respective null-segregants when grown in the growth chamber, and a 13–29% higher leaf FA in field mini swards, with no significant treatment effect observed between E + or E- individuals. Similarly, field grown AR37-infected progeny achieved increases of 27–33% for hemizygous DGAT + CO, and 38–58% for homozygous DGAT + CO, depending on harvest. The incremental elevation of FA observed from hemizygous to homozygous DGAT + CO progeny corresponds with an increase in gene dosage. This relationship between transgene dosage with gene expression and phenotype has been reported in other species such as white clover [29], rice [30] or maize [31, 32], and is similar to that reported by Cooney et al. [14] where increases in accumulation of FA were observed in high-copy number DGAT + CO primary transgenic lines (T_0_).

Increased biomass yields have been reported both for DGAT + CO ryegrass and Arabidopsis when grown in a spaced pot arrangement, with the increase in growth positively correlated to the increased levels of accumulated FA [11, 13, 14, 28]. Additionally, Badenhorst et al. [33] identified increased yield of biomass for T_0_ plants with an altered fructosyl-transferase (designed to increase water soluble carbohydrates levels) grown as spaced plant in the field. However, at least for DGAT + CO, this growth advantage is not maintained when plants are grown as a dense canopy in the field where herbage growth rates for DGAT + CO were similar to the comparative null-segregants across two field seasons [28]. In this current experiment we measured relative growth rates and herbage yield in the growth chamber and field respectively. For plants infected with AR1, we did not identify differences in biomass production. This suggests that presence of *Epichloë in planta* is not detrimental to performance of the DGAT + CO trait either in terms of plant growth or FA accumulation.

### Endophyte interactions

Quantitative trait locus analysis has demonstrated that the endophyte phenotype is regulated by host genetics [34, 35] so we considered that introduction of DGAT + CO and subsequent accumulation of FA in foliar tissues could negatively impact the grass-endophyte association. Also, in contrast to the apical extension model of hyphal growth observed in most fungal species, whereby filamentous fungi grow only through extension at the hyphal tip, the hyphae of *Epichloë* spp. within infected grass leaves are attached to the grass host cells allowing the fungus to extend at the same rate as the growing leaf, through a mechanism of intercalary division and extension [22]. As such, disruption of *Epichloë* mycelial growth resulting from FA accumulation could subsequently limit systemic colonisation of the endophyte from tiller to tiller, or transmission vertically through the seed from one plant generation to the next, both of which are requisite to the maintenance of a successful ryegrass-*Epichloë* association. In this experiment, *Epichloë* infection in a DGAT + CO genetic background with enhanced FA accumulation does not interrupt this novel mechanism of hyphal growth. Also, vertical transmission from T_2_ plants into the T_3_ generation was not limited by DGAT + CO, where up to 100% transmission was observed in both parents across several reciprocal crosses.

The AR1 strain was initially selected for this study as it possesses a reliably consistent high level of vertical transmission frequency across a wide range of germplasm and has been marketed commercially since 2001 [36]. By contrast, vertical transmission of AR37 has been found to be heavily influenced by the genetics of the ryegrass host. Three populations subjected to recurrent selection for AR37 transmission demonstrated high transmission frequencies (88, 92 and 93%), while lower transmission frequencies were observed in two populations developed without selection (69 and 70%) [24]. Therefore, molecular breeding strategies designed to stabilise transgenic traits in an obligate outcrossing species, where each seed represents an individual genotype as outlined by Badenhorst et al*.* [37], will also need to be cognisant of maintaining high rates of AR37 transmission through this process.

Similar reductions in endophyte-derived alkaloid concentrations, to those observed in endophyte infected DGAT + CO, have been described in previous experiments which measured endophyte-derived alkaloid concentration and mycelial biomass in perennial ryegrass cultivars with elevated levels of water-soluble carbohydrate (WSC) developed with traditional breeding techniques or from populations using recombinant technologies [38, 39]. Using two perennial ryegrass cultivars differing in carbohydrate content infected with three *Epichloë* strains (AR1, AR37, and a common-toxic strain) Rasmussen et al*.* [39] identified that endophyte concentration and alkaloid production in the high sugar cultivar ‘AberDove’ was reduced by 50% and by 40% under high nitrogen supply, and that the combined effects had a 75% reduction. By contrast, the correlation between a reduction in mycelial mass and alkaloid concentration was not observed in DGAT + CO infected either with AR1 or AR37. In a similar set of experiments, Giraldo et al*.* [38] examined the interaction of secondary metabolite production in plants expressing a chimeric fusion of sucrose: sucrose 1-fructosyltransferase and fructosyltransferase genes (SST-FFT), to produce high levels of WSC. Clones of a T_0_ founder plant from a single transformation event were used to generate seed derived from a range of cultivars infected with various *Epichloë* strains in an experiment designed to determine if insertion, or expression, of SST-FFT altered the toxicity of the endophyte-grass association. A significant difference in alkaloid concentration was detected between SST-FFT and the null-segregant progeny in 5 of 28 combinations investigated. Peramine was reduced by 62% and 61% in two of the associations with cultivar ‘Trojan’ infected with strain NEA6 and cultivar ‘Alto’ infected with a common-toxic strain, respectively, however, no statistically significant differences in epoxyjanthitrem concentrations were identified for any of the cultivar combinations [38]. Nonetheless, stability of the SST-FFT phenotype (WSC and biomass) following introduction of the endophyte into this germplasm was not reported.

*Epichloë*-derived alkaloids often accumulate to a higher concentration within endophyte infected grass seed than in vegetative tissue which is thought to be a strategy utilised by the plant host to defend against insect predation. The subsequent translocation of the stored alkaloid into seedlings during germination acts as a deterrent largely to insect herbivory during plant establishment [40, 41]. This initial high level of alkaloid concentration in young seedlings provides the capacity for good insect resistance but is followed by a period of reduction which leaves seedlings vulnerable to insect predation until growth of the endophyte within the developing plants allows sufficient de novo alkaloid synthesis [42]. It has been postulated that a decreasing supply of substrate due to plant proliferation results in a constraint on secondary metabolite synthesis in the early stage of plant development [43]. Consistent with the previous reports, we observed a 2–threefold increase of epoxyjanthitrem concentrations in seed compared to leaf tissues in both the null-segregant and homozygous DGAT + CO infected with AR37. In addition, a 25% reduction in epoxyjanthitrems was measured in homozygous DGAT + CO seed compared to the null-segregant, but this remains within the range previously measured in the commercial cultivar Ceres One50 [43]. The percentage alkaloid reduction measured in homozygous DGAT + CO seed was similar to field harvested leaf material.

Rasmussen et al*.* [44] identified that metabolic traits and nutrient availability are critical factors in determining metabolic and physiological outcomes of the grass-endophyte association. In contrast to the high-WSC material described in those previous studies, a partial depletion in leaf WSC commensurate with the increase in FA has been described for DGAT + CO events when grown in controlled temperature chambers [14] but this did not translate to a field environment [28]. Rasmussen et al*.* [39] determined that quantification of both epoxyjanthitrems and peramine was linearly related to endophyte mycelial biomass, however, the reductions in mycelial mass which were observed between DGAT + CO and the null-segregant were not significant. There were significant reductions in peramine detected only in leaf tissues harvested from DGAT + CO plants in the growth chamber and Harvest 5 in the field, while epoxyjanthitrem concentrations were lower only in homozygous DGAT + CO plants from Harvest 4. This suggests that mechanisms other than carbohydrate exchange, as proposed by Rasmussen et al*.* [45], may be influential in maintaining endophyte-derived alkaloid levels within *Epichloë*-perennial ryegrass associations with DGAT + CO. For instance, Giraldo et al*.* [38] proposed that differences in endophyte-derived alkaloids detected in the high WSC transgenic plants might not be related to alkaloid biosynthesis but may result from a phenomenon known as the “dilution effect” first described by Lane and Christensen [46]. This can be most succinctly described as environmental factors stimulating plant growth in excess of endophyte growth. Alternatively, carbon sequestration may be a limiting factor in DGAT + CO where accumulated FA are encapsulated in an inaccessible form since the endophyte is solely dependent upon their hosts for energy and carbon supply.

In addition to host genetics, endophyte characteristics such as vertical transmission frequency, mycelial biomass and alkaloid concentration vary on a seasonal basis [47–49]. Maintaining bioactive levels of endophyte-derived metabolites are important in perennial ryegrass pastures with low alkaloid concentrations since plants are potentially highly susceptible to insect predation in many localities. Typically, endophyte-derived alkaloid concentrations are lowest during winter and spring, with the highest concentrations accruing in the summer [50, 51]. Alkaloid levels measured in field grown DGAT + CO appear to be broadly similar to those reported in farm studies comparing the effects of endophyte strains on ryegrass pastures in Australia and New Zealand [51–53]. A threshold of 15 to 20 µg/g peramine is recommended to provide robust resistance to Argentine stem weevil in New Zealand for AR1 infected pastures [54]. For AR37, significant feeding inhibition was detected for porina larvae (*Wiseana cervinata*), a common pest of New Zealand pastures, at total epoxyjanthitrem concentrations as low as 14 µg/g [55] with peaks between 20 to 50 µg/g measured for mid-summer timed harvests [42, 53]. The peramine concentrations for the null-segregants were within the expected range for plants grown in the field while the reduced concentration observed for DGAT + CO was slightly lower than the suggested threshold, although this difference was only statistically significant for the fifth harvest and was within the range measured for whole tillers harvested from irrigated pastures in Australia [56]. Epoxyjanthitrem concentrations for DGAT + CO were within the bioactive range and similar to those reported for AR37-infected pastures in New Zealand and Australia respectively [50, 53] but they were somewhat lower than those reported elsewhere for New Zealand pastures [51, 57].

Multi-year, multi-site experiments in an Australasian pasture setting where DGAT + CO ryegrass is likely to be deployed will be critical for assessing genotype × environment effects, which are a major influence on phenotype in this species. Furthermore, they would provide a useful addition to the data reported here given the seasonal and year to year fluctuations in endophyte-derived alkaloid concentrations. However, the changes in alkaloid concentrations observed in these experiments with DGAT + CO did not alter the toxicity of the endophyte-grass association and is similar to changes previously reported by Giraldo et al. [38]. Future breeding steps for introgression of DGAT + CO into elite material, coordinated with careful selection of germplasm known to increase alkaloid levels could be used to offset any decreases seen due to the DGAT + CO trait.

## Conclusion

Our measurements indicate that presence of the endophyte does not negatively impact plant growth or the DGAT + CO trait, which has been engineered to accumulate fatty acids in foliar tissues to increase the metabolizable energy available to grazing ruminants. While high rates of vertical transmission were observed for both endophyte strains used in these experiments and no significant reduction in mycelial biomass identified, a 30–40% reduction in endophyte-derived alkaloid concentration occurred both in growth chamber and for two harvests of the field grown material. From an agronomic perspective, using DGAT + CO to enhance the GE of pastures by upwards of 0.5 kJ/gDW has great potential but, for the technology to have on-farm impact, will need to be incorporated into elite germplasm with the widespread industry practice of infection with strains of *Epichloë* endophyte. Therefore, further investigation is warranted to elucidate the interactions between the DGAT + CO trait and *Epichloë* endophytes in perennial ryegrass given that alkaloid concentration is known to vary depending on climatic and seasonal fluctuations, location, host genetics and the interaction amongst those factors. Multi-site and multi-season trials (including insect testing) conducted in temperate environments suited to ryegrass production under standard pastoral grazing system management would be a useful validation of current results and accurately quantify metabolizable energy and reduction in methane emissions if combined with animal feeding trails.

## Materials and methods

### Plant material

The DGAT + CO trait results from over-expression of DGAT1 and cys-OLE genes under the expression of the rice RbcS and CAB promoters respectively (Supplementary Figure S2). Two T_2_ populations of perennial ryegrass, segregating for the DGAT + CO expression cassette, were used to compare DGAT + CO and *Epichloë* characteristics of strains AR1 and AR37. A diagrammatic representation of the crosses required for development of T_2_ populations infected (E +) or not infected (E-) with endophyte is shown in Supplementary Figure S3. The population colonised by AR1 was produced by crossing clones of ODR4501 [28], a primary event (T_0_) obtained by microprojectile bombardment, separately with 16 genotypes from the cultivar Bronsyn with T_1_ seed collected from the transgenic parent. Subsequently, 22 T_1_ progeny from ODR4501/Bronsyn were pair-crossed to a genotype from cultivar Alto infected with AR1. The T_2_ population originated from seed collected from both the AR1-infected parent and the DGAT + CO T_1_ parent to give a population comprising of both E + and E-along with DGAT + CO and null-segregant progeny (i.e., DGAT + CO/E + , DGAT + CO/E-, null/E + , null/E-). Seed families obtained from these crosses were used for growth chamber and 2019 field experiments. Similarly, the AR37-infected perennial ryegrass population was produced by crossing clones of RCR5101 [14], primary event (T_0_) obtained by agrobacterium-mediated transformation, separately with three discrete genotypes from proprietary breeding pools infected with AR37. T_1_ seed were collected from the (AR37-infected) non-transgenic parent. Subsequently, 6 T_1_ progeny from each selection were inter-crossed with DGAT + CO progeny from each of the other two selections to give an AR37-infected T_2_ population segregating 1:2:1 (25% homozygous DGAT + CO, 50% hemizygous DGAT + CO and 25% null). Seed obtained from these crosses were used for 2021 field experiments.

To measure vertical transmission frequency from maternal parent plants to seedlings, four T_2_ seedlings infected with AR1 were crossed pairwise to AR1-infected genotypes of cultivar Ceres One50 and seed collected from both parents. Between 13–18 T_3_ progeny collected from crosses were germinated from each parent and grown under natural daylength until the three-tiller stage whereupon one tiller from each plant was harvested for tissue print-immunoassay for the presence of endophyte [58].

### Transgene status identification

Transgene status of germinated seedlings in the AR1 infected T_2_ population was determined by leaf immunoblot for the oleosin protein following the method described in Beechey-Gradwell *et. al* [28]*.* and the resulting chemiluminescence was visualised using a Bio-Rad ChemiDoc™ XRS^+^ imaging system.

The zygosity of seedlings in the AR37-infected T_2_ population was determined by ddPCR as described by Xu [59] using Copy Number Variation Analysis (Bio-Rad 2017). The *hph* selectable marker gene was used as the target gene and the ryegrass *GIGANTEA* gene as the reference (*LpGI*, GenBank: DQ534010). The target and reference genes were amplified from 60 ng of genomic DNA using TaqMan hydrolysis probes in the ddPCR assay and data analysed with QuantaSoft software versions 1.3.2.0 (Bio-Rad Laboratories).

### Assessment of endophyte colonisation using confocal microscopy

Twenty T_2_ progeny collected from each parent of a pairwise cross were first assayed by immunoblot to determine the presence of endophyte and oleosin expression. After identifying a 100% transmission frequency from the AR1-infected parent and the expected 1:1 segregation of the transgene in these populations, we examined DGAT + CO and null-segregant progeny to determine which were E + or E-. Confocal microscopy was then used to determine the distribution of endophyte hyphae within vegetative tillers of greenhouse grown plants. Tiller segments from 12 plants (6 DGAT + CO and 6 null) were hand-sectioned longitudinally. Hyphae were stained with aniline blue diammonium salt (Sigma Chemicals Co., Italy) using a method adapted from Becker *et. al* [60]*.* by soaking tissues overnight in a refrigerator in 0.02% aniline blue (in PBS) and Alexa Fluor 488 (ng/mL) was used to stain the hyphal septa. Sections were observed under an inverted Olympus FV10i-w confocal laser scanning microscope (Tokyo, Japan). Excitation of aniline blue was at 405 nm with detection using a 420–460 nm barrier filter and excitation of Alexa Fluor 488 at 437 nm with detection using 490–590 nm barrier filter.

### Assessment of endophyte infection frequency using immuno-detection

Plants were grown to the 3–4 tiller stage prior to assessment of endophyte infection frequency. Tillers were cut basally approximately 5 mm from potting mix level and the freshly cut end pressed onto nitrocellulose membrane (Amersham Protran 0.45 μm NC). Tillers from plants of known endophyte status (E + or E-) were blotted to the membrane as controls. Blotted membranes were then assayed for the presence of *Epichloë* using an immunoblotting technique utilising polyclonal rabbit antibodies raised against homogenized mycelium of *E. festucae* var. *lolii* strain Lp5 [58].

### Quantification of Epichloë mycelial biomass and indole diterpene alkaloids

Twenty milligrams of freeze-dried and ball milled plant material was extracted in a PBST solution for calculation of *Epichloë* mycelial biomass by the competitive ELISA method described by Faville et al*.* [34] using an antigen previously prepared from *E. festucae* var. *lolii* mycelium following the method of Ball et al*.* [47]. Samples were analysed for indole diterpenes and peramine as per the method of Lukito et al*.* [61]. Briefly, lyophilised and ground plant samples (50 ± 5 mg) were extracted with 80% MeOH (1 mL, containing internal standards) for 1 h. After centrifuging, supernatants were analysed by LC–MS/MS. Peramine was quantified using homoperamine as an internal standard, while the epichloëcyclin F and oxo-epichloëcyclin F were normalised to homoperamine, and the indole diterpenes peak areas were normalised to a paxilline external standard. This normalisation allows comparison between samples for an indole diterpene but does not allow comparison between indole diterpenes within a sample. Epoxyjanthitrems were quantified using a reference material of known concentration. Mycelial mass is reported as mg/g DW while alkaloid measurements are presented as mean alkaloid concentration in μg/g DW.

### Relative growth rates of perennial grass genotypes

Four genotypes representing DGAT + CO/E + , DGAT + CO/E-, null/E + , and null/E- were selected and propagated via the production of clonal ramets as described by Beechey-Gradwell et al*.* [13]. Briefly, triplicate clones of each genotype were propagated as five tiller ramets and these underwent three rounds of propagation at 4 weekly intervals to synchronise tiller development. All plants were grown in a controlled temperature chamber with ~ 600 µmol photons m^2^ s^-1^ red/blue light provided by 600 W NanoPro LED lights (LED Grow Lights, New Zealand), 20 °C/15 °C day/night temperature and 12-h photoperiod.

At completion of the propagation phase, two comparable five-tiller ramets were selected from the replicates of each genotype. The first was harvested to determine the starting weight while the other was transplanted into pots containing 1.3 L sand and flushed thrice weekly with 100 mL of basal nutrient solution described in Andrews [62], containing 4 mM KNO_3_. Plants were maintained in a closely packed arrangement during this growth phase. Five weeks after propagation, plants were harvested, oven dried and the relative growth rate (RGR) was calculated as described by Poorter [63]: RGR = (ln W_2_ – ln W_1_)/(t_2_ – t_1_) where W_1_ = post-establishment dry weight, W_2_ = final harvest dry weight, t_1_ = day 0, and t_2_ = day 35.

### Fatty acid analysis

Leaf material was harvested and freeze dried prior to grinding in a Tissuelyser bead mill (Qiagen). A 10 mg aliquot of ground material was sampled per plant and FA extracted in hot methanolic HCl modified after Browse et al*.* [64]. FA were quantified by GC–MS (QP 2010 SE, Shimadzu Corp., Kyoto, Japan) against an internal standard of 10 mg C15:0 and total FA was calculated as the sum of palmitic acid (16:0), palmitoleic acid (16:1), stearic acid (18:0), oleic acid (18:1), linoleic acid (18:2) and linolenic acid (18:3).

### Field site

Field experiments were conducted from May to October in 2019 with populations associated with AR1 and 2021 with populations associated with AR37 at the Greenley Research Centre, Novelty, MO, USA (40.0216° N, 92.1903° W). Climatic data for these periods (average and maximum daily temperatures, solar radiation, and precipitation) is available (http://agebb.missouri.edu/weather/realtime/novelty.asp). The soil type was Putnam silt loam (fine, smectitic, mesic Vertic Albaqualfs) that had been removed from a corn (*Zea mays* L.), soybean (*Glycine max* [L.] Merr.) rotation in May 2017. Herbicide application (pendimethalin (N-(1-ethylpropyl)-3,4-dimethyl-2,6-dinitrobenzenamine) broadcast at 1.07 kg/ha) or mechanical tillage were used to remove Volunteer plants in the plot area and 58 m distance from the plot. The plot was enclosed by deer (*Cervidae*) netting to protect plants from vertebrate browsers. Prior to planting the plot was fertilized with 34 kgN ha^−1^ as urea (SuperU, Koch Agronomic Services, Wichita, USA). For the rest of the trial, 34 kgN ha^−1^ fertilizer applications were made within a day following each harvest. The plot was irrigated as required during the experimental period.

Seeds were germinated on moist sand in seedling trays under LED lights, moved to a glasshouse and regularly watered with half-strength Hoagland’s nutrient solution for approximately 2 months before transplanting into field swards. Treatments (DGAT + CO/E + , DGAT + CO/E-, null/E + , null/E- for AR1 and homozygous DGAT + CO/E + , hemizygous DGAT + CO/E + or null DGAT + CO/E + for AR37) were randomly assigned to sward plots consisting of 5 rows of 5 plants each, with 10 cm spacing between plants within a row, and 20 cm spacing between rows. Only the centre 9 plants were analysed, while the outer sixteen were treated as border plants and were not analysed. Treatments with AR1 infected plants were planted in 2019 and limited to 1 sward per treatment, whereas AR37 infected plants were planted in 2021 and comprised 7 swards per treatment. A total of five harvests took place, to a defoliation height of 5 cm. Harvest 1 occurred 38 days after transplanting into the field with a regrowth interval of between 22 and 29 days for subsequent harvests. FA and endophyte-derived alkaloid concentrations were measured in harvests 3 to 5 in the AR1-infected treatments and harvests 4 and 5 in AR37-infected treatments.

### Statistical analysis

Statistical analysis was performed in R version 4.02 [65]. All models except for the FA and RGR data sets were analysed using a linear mixed model using the lme4 package version 1.1–25 [66] with trait and either harvest or tissue as the fixed effects and plant as the random effect. Linear models were used for the FA and RGR data sets with trait as the fixed effect. Post-hoc tests were generated using the emmeans R package version 1.5.0 [67] with *P*-value multiple-testing adjustments done using Tukey’s method where *p*-values less than 0.05 were considered significant.

### Supplementary Information

**Additional file 1: Supplementary Table S1.** Fatty acid profiles in DGAT + CO and null plants grown in the field. **Supplementary Figure S1.** Herbage yield of DGAT + CO T_2_ and nulls grown in field. **Supplementary Figure S2.** Binary vector map of the DGAT + CO expression construct. **Supplementary Table S2.** Full *in planta* alkaloid analysis of endophyte infected DGAT + CO. **Supplementary Figure S3.** Diagrammatic representation of the crosses for developing T_2_ populations.

## Data Availability

The datasets analysed during the current study are available from the corresponding author on request.

## Abbreviations

DGAT + CO: Combined expression in green tissues of a diacylglycerol O-transferase 1 and a cysteine oleosin gene
FA: Fatty acid
AR1: *E. festucae* Var. *lolii* strain AR1
AR37: *Epichloë* Sp. LpTG-3 strain AR37
E-: Endophyte free
E +: Endophyte infected
RGR: Relative growth rate
LC–MS/MS: Liquid chromatography coupled to tandem mass spectrometry
DW: Dry weight
GE: Gross energy
WSC: Water soluble carbohydrate
SST-FFT: A fusion of sucrose: sucrose 1-fructosyltransferase and fructosyltransferase genes

## References

[1] Humphreys M, Feuerstein U, Vandewalle M, Baert J. Ryegrasses. In: Fodder crops and amenity grasses. New York: Springer; 2010. p. 211–260.

[2] Chapman DF, Ludemann CI, Wims CM, Kuhn-Sherlock B (2023). The contribution of perennial ryegrass (Lolium perenne L.) breeding to whole pasture productivity under dairy cattle grazing in New Zealand. 2. Rates of gain in production traits and economic value. Grass Forage Sci.

[3] Chapman DF, Wims CM, Ludemann CI, Kuhn-Sherlock B (2023). The contribution of perennial ryegrass (Lolium perenne L.) breeding to whole pasture productivity under dairy cattle grazing in New Zealand. 1. Variation in yield, nutritive value and persistence-related traits. Grass Forage Sci.

[4] Barrett BA, Faville MJ, Nichols SN, Simpson WR, Bryan GT, Conner AJ (2015). Breaking through the feed barrier: options for improving forage genetics. Anim Prod Sci.

[5] Cen H, Ye W, Liu Y, Li D, Wang K, Zhang W (2016). Overexpression of a Chimeric Gene, OsDST-SRDX, improved salt tolerance of perennial ryegrass. Sci Rep.

[6] Patel M, Milla-Lewis S, Zhang W, Templeton K, Reynolds WC, Richardson K, Biswas M, Zuleta MC, Dewey RE, Qu R (2015). Overexpression of ubiquitin-like LpHUB1 gene confers drought tolerance in perennial ryegrass. Plant Biotechnol J.

[7] Xu J, Schubert J, Altpeter F (2001). Dissection of RNA-mediated ryegrass mosaic virus resistance in fertile transgenic perennial ryegrass (Lolium perenne L.). Plant J.

[8] Panter S, Mouradov A, Badenhorst P, Martelotto L, Griffith M, Smith KF, Spangenberg G (2017). Re-programming photosynthetic cells of perennial ryegrass (Lolium perenne L) for fructan biosynthesis through transgenic expression of fructan biosynthetic genes under the control of photosynthetic promoters. Agronomy.

[9] Faville MJ, Richardson K, Gagic M., Mace W, Sun XZ, Harrison S, Knapp K, Jahufer MZZ , Palinisamy R, Pirlo S, Johnson R, Rasmussen S, Bryan G. Genetic improvement of fibre traits in perennial ryegrass. Proceedings of the New Zealand Grassland Association 2010, 72:71-78.

[10] Winichayakul S, Cookson R, Scott R, Zhou J, Zou X, Roldan M, Richardson K, Roberts N (2008). Delivery of grasses with high levels of unsaturated, protected fatty acids. Proceedings of the New Zealand Grassland Association.

[11] Winichayakul S, Scott RW, Roldan M, Hatier JH, Livingston S, Cookson R, Curran AC, Roberts NJ (2013). In vivo packaging of triacylglycerols enhances Arabidopsis leaf biomass and energy density. Plant Physiol.

[12] Rasmussen J, Harrison A. The benefits of supplementary fat in feed rations for ruminants with particular focus on reducing levels of methane production. Int Sch Res Notices. 2011. 10.5402/2011/613172. 10.5402/2011/613172 PMC3658489 23738103

[13] Beechey-Gradwell Z, Cooney L, Winichayakul S, Andrews M, Hea SY, Crowther T, Roberts N (2020). Storing carbon in leaf lipid sinks enhances perennial ryegrass carbon capture especially under high N and elevated CO2. J Exp Bot.

[14] Cooney LJ, Beechey-Gradwell Z, Winichayakul S, Richardson KA, Crowther T, Anderson P, Scott RW, Bryan G, Roberts NJ (2021). Changes in leaf-level nitrogen partitioning and mesophyll conductance deliver increased photosynthesis for Lolium Perenne Leaves Engineered to accumulate lipid carbon sinks. Front Plant Sci.

[15] Winichayakul S, Beechey-Gradwell Z, Muetzel S, Molano G, Crowther T, Lewis S, Xue H, Burke J, Bryan G, Roberts NJ (2020). In vitro gas production and rumen fermentation profile of fresh and ensiled genetically modified high-metabolizable energy ryegrass. J Dairy Sci.

[16] Caradus JR, Card SD, Hewitt KG, Hume DE, Johnson LJ (2021). Asexual Epichloë Fungi—Obligate Mutualists. Encyclopedia.

[17] Johnson LJ, Caradus JR (2019). The science required to deliver Epichloë endophytes to commerce. Endophytes for a growing world.

[18] Easton H, Fletcher L (2006). The importance of endophyte in agricultural systems-changing plant and animal productivity. NZGA Res Pract Series.

[19] Johnson LJ, De Bonth ACM, Briggs LR, Caradus JR, Finch SC, Fleetwood DJ, et al. The exploitation of epichloae endophytes for agricultural benefit. Fungal Divers. 2013;60(1):171–88.

[20] Caradus JR, Card SD, Finch SC, Hume DE, Johnson LJ, Mace WJ, Popay AJ (2022). Ergot alkaloids in New Zealand pastures and their impact. N Z J Agric Res.

[21] Johnson LJ, Bastías DA, Caradus JR, Chettri P, Forester NT, Mace WJ, et al. The dynamic mechanisms underpinning symbiotic Epichloë–grass interactions: Implications for sustainable and resilient agriculture. In: Microbiome Stimulants for Crops. Cambridge: Elsevier; 2021. p. 73–108.

[22] Christensen MJ, Bennett RJ, Ansari HA, Koga H, Johnson RD, Bryan GT, Simpson WR, Koolaard JP, Nickless EM, Voisey CR (2008). Epichloe endophytes grow by intercalary hyphal extension in elongating grass leaves. Fungal Genet Biol.

[23] Zhang W, Card SD, Mace WJ, Christensen MJ, McGill CR, Matthew C (2017). Defining the pathways of symbiotic Epichloë colonization in grass embryos with confocal microscopy. Mycologia.

[24] Gagic M, Faville MJ, Zhang W, Forester NT, Rolston MP, Johnson RD, Ganesh S, Koolaard JP, Easton HS, Hudson D (2018). Seed Transmission of Epichloe Endophytes in Lolium perenne Is Heavily Influenced by Host Genetics. Front Plant Sci.

[25] Zhang W, Forester NT, Moon CD, Maclean PH, Gagic M, Arojju SK, Card SD, Matthew C, Johnson RD, Johnson LJ (2022). Epichloë seed transmission efficiency is influenced by plant defense response mechanisms. Front Plant Sci.

[26] Giraldo PA, Shinozuka H, Spangenberg GC, Cogan NOI, Smith KF (2019). Safety assessment of genetically modified feed: is there any difference from food?. Front Plant Sci.

[27] Caradus JR. Intended and unintended consequences of genetically modified crops–myth, fact and/or manageable outcomes? New Zealand J Agric Res. 2022;66(6):519–619.

[28] Beechey-Gradwell Z, Kadam S, Bryan G, Cooney L, Nelson K, Richardson K, Cookson R, Winichayakul S, Reid M, Anderson P (2022). Lolium perenne engineered for elevated leaf lipids exhibits greater energy density in field canopies under defoliation. Field Crop Res.

[29] Roldan MB, Cousins G, Muetzel S, Zeller WE, Fraser K, Salminen J-P, et al. Condensed tannins in white clover (Trifolium repens) foliar tissues expressing the transcription factor TaMYB14-1 bind to forage protein and reduce methane emissions in vitro. Front Plant Sci. 2022. 10.3389/fpls.2021.777354. 10.3389/fpls.2021.777354 PMC8774771 35069633

[30] James V, Avart C, Worland B, Snape J, Vain P (2002). The relationship between homozygous and hemizygous transgene expression levels over generations in populations of transgenic rice plants. Theor Appl Genet.

[31] Eghrari K, de Brito AH, Baldassi A, Balbuena TS, Fernandes OA, Môro GV (2019). Homozygosis of Bt locus increases Bt protein expression and the control of Spodoptera frugiperda (Lepidoptera: Noctuidae) in maize hybrids. Crop Prot.

[32] Hood EE, Devaiah SP, Fake G, Egelkrout E, Teoh K, Requesens DV, Hayden C, Hood KR, Pappu KM, Carroll J (2012). Manipulating corn germplasm to increase recombinant protein accumulation. Plant Biotechnol J.

[33] Badenhorst PE, Panter S, Palanisamy R, Georges S, Smith KF, Mouradov A, Mason J, Spangenberg GC (2018). Molecular breeding of transgenic perennial ryegrass (Lolium perenne L.) with altered fructan biosynthesis through the expression of fructosyltransferases. Mol Breed.

[34] Faville MJ, Briggs L, Cao M, Koulman A, Jahufer MZ, Koolaard J, Hume DE (2015). A QTL analysis of host plant effects on fungal endophyte biomass and alkaloid expression in perennial ryegrass. Mol Breed.

[35] Easton H, Latch G, Tapper B, Ball OP (2002). Ryegrass host genetic control of concentrations of endophyte-derived alkaloids. Crop Sci.

[36] Popay AJ, Hume DE (2020). Endophytes for improving ryegrass performance: Current status and future possibilities.

[37] Badenhorst PE, Smith KF, Spangenberg G (2016). Development of a molecular breeding strategy for the integration of transgenic traits in outcrossing perennial grasses. Agronomy.

[38] Giraldo PA, Elliott C, Badenhorst P, Kearney G, Spangenberg GC, Cogan NOI, Smith KF (2018). Evaluation of endophyte toxin production and its interaction with transgenic perennial ryegrass (Lolium perenne L.) with altered expression of fructosyltransferases. Transgenic Res.

[39] Rasmussen S, Parsons AJ, Bassett S, Christensen MJ, Hume DE, Johnson LJ, Johnson RD, Simpson WR, Stacke C, Voisey CR (2007). High nitrogen supply and carbohydrate content reduce fungal endophyte and alkaloid concentration in Lolium perenne. New Phytol.

[40] Ball O, Prestidge R, Sprosen J. Effect of plant age and endophyte viability on peramine and lolitrem B concentration in perennial ryegrass seedlings. In: Proceedings of the 2nd international symposium on Acremonium/grass interactions. Palmerston North: Grasslands Research Centre Palmerston North; 1993. p. 63–66.

[41] Ball O-P, Barker G, Prestidge R, Sprosen J (1997). Distribution and accumulation of the mycotoxin lolitrem B in Neotyphodium lolii-infected perennial ryegrass. J Chem Ecol.

[42] Ruppert KG, Matthew C, McKenzie CM, Popay AJ (2017). Impact of Epichloë endophytes on adult Argentine stem weevil damage to perennial ryegrass seedlings. Entomol Exp Appl.

[43] Hewitt KG, Mace WJ, McKenzie CM, Matthew C, Popay AJ (2020). Fungal alkaloid occurrence in endophyte-infected perennial ryegrass during seedling establishment. J Chem Ecol.

[44] Rasmussen S, Parsons AJ, Fraser K, Xue H, Newman JA (2008). Metabolic profiles of Lolium perenne are differentially affected by nitrogen supply, carbohydrate content, and fungal endophyte infection. Plant Physiol.

[45] Rasmussen S, Liu Q, Parsons AJ, Xue H, Sinclair B, Newman JA (2012). Grass-endophyte interactions: a note on the role of monosaccharide transport in the Neotyphodium lolii-Lolium perenne symbiosis. New Phytol.

[46] Lane GA, Christensen MJ. Coevolution of Fungal Endophytes with Grasses: The Signifi-cance of Secondary Metabolites. In: Microbial endophytes. New York: Marcel Dekker: CRC Press; 2000. p. 355–402.

[47] Ball O, Prestidge R, Sprosen J (1995). Interrelationships between Acremonium lolii, peramine, and lolitrem B in perennial ryegrass. Appl Environ Microbiol.

[48] Freitas PP, Hampton JG, Rolston MP, Glare TR, Miller PP, Card SD (2020). A tale of two grass species: Temperature affects the symbiosis of a mutualistic Epichloë endophyte in both tall fescue and perennial ryegrass. Front Plant Sci.

[49] Hennessy LM, Popay AJ, Finch SC, Clearwater MJ, Cave VM (2016). Temperature and plant genotype alter alkaloid concentrations in ryegrass infected with an Epichloë endophyte and this affects an insect herbivore. Front Plant Sci.

[50] Fletcher L, Finch S, Sutherland B, deNicolo G, Mace W, Van Koten C, Hume D (2017). The occurrence of ryegrass staggers and heat stress in sheep grazing ryegrass-endophyte associations with diverse alkaloid profiles. N Z Vet J.

[51] Thom E, Popay A, Waugh C, Minneé E (2014). Impact of novel endophytes in perennial ryegrass on herbage production and insect pests from pastures under dairy cow grazing in northern New Zealand. Grass Forage Sci.

[52] Bluett S, Thom E, Clark D, Macdonald K, Minneé E (2005). Effects of perennial ryegrass infected with either AR1 or wild endophyte on dairy production in the Waikato. N Z J Agric Res.

[53] Moate P, Williams S, Grainger C, Hannah M, Mapleson D, Auldist M, Greenwood J, Popay A, Hume D, Mace W (2012). Effects of wild-type, AR1 and AR37 endophyte-infected perennial ryegrass on dairy production in Victoria, Australia. Anim Prod Sci.

[54] Popay A, Hume D, Davis K, Tapper B (2003). Interactions between endophyte (Neotyphodium spp.) and ploidy in hybrid and perennial ryegrass cultivars and their effects on Argentine stem weevil (Listronotus bonariensis). New Zealand J Agric Res.

[55] Finch SC, Prinsep MR, Popay AJ, Wilkins AL, Webb NG, Bhattarai S, Jensen JG, Hawkes AD, Babu JV, Tapper BA (2020). Identification and structure elucidation of epoxyjanthitrems from Lolium perenne infected with the endophytic fungus Epichloë festucae var. lolii and determination of the tremorgenic and anti-insect activity of epoxyjanthitrem I. Toxins.

[56] Lowe KF, Bowdler TM, Hume DE, Casey ND, Tapper BA (2008). The effect of endophyte on the performance of irrigated perennial ryegrasses in subtropical Australia. Aust J Agric Res.

[57] Finch S, Fletcher L, Babu J (2012). The evaluation of endophyte toxin residues in sheep fat. N Z Vet J.

[58] Simpson W, Schmid J, Singh J, Faville M, Johnson R (2012). A morphological change in the fungal symbiont Neotyphodium lolii induces dwarfing in its host plant Lolium perenne. Fungal Biol.

[59] Xu X, Peng C, Wang X, Chen X, Wang Q, Xu J (2016). Comparison of droplet digital PCR with quantitative real-time PCR for determination of zygosity in transgenic maize. Transgenic Res.

[60] Becker Y, Eaton CJ, Brasell E, May KJ, Becker M, Hassing B, Cartwright GM, Reinhold L, Scott B (2015). The Fungal Cell-Wall Integrity MAPK Cascade Is Crucial for Hyphal Network Formation and Maintenance of Restrictive Growth of Epichloe festucae in Symbiosis With Lolium perenne. Mol Plant Microbe Interact.

[61] Lukito Y, Chujo T, Hale TK, Mace W, Johnson LJ, Scott B (2019). Regulation of subtelomeric fungal secondary metabolite genes by H3K4me3 regulators CclA and KdmB. Mol Microbiol.

[62] Andrews M, Love B, Sprent J (1989). The effects of different external nitrate concentrations on growth of Phaseolus vulgaris cv. Seafarer at chilling temperatures. Ann Appl Biol.

[63] Poorter H (1989). Plant growth analysis: towards a synthesis of the classical and the functional approach. Physiol Plant.

[64] Browse J, McCourt PJ, Somerville CR (1986). Fatty acid composition of leaf lipids determined after combined digestion and fatty acid methyl ester formation from fresh tissue. Anal Biochem.

[65] Team RC: R: A Language and Environment for Statistical Computing. R Foundation for Statistical Computing, Vienna, Austria. 2023. https://www.R-project.org/.

[66] Bates D, Mächler M, Bolker B, Walker S. Fitting linear mixed-effects models using lme4. arXiv preprint arXiv:14065823. 2014.

[67] Lenth R, Singmann H, Love J, Buerkner P, Herve M. R. Package ‘emmeans’. Am Stat. 34:2216-221.

