# Supplementary material for: Epichloë fungal endophyte interactions in perennial ryegrass (Lolium perenne L.) modified to accumulate foliar lipids for increased energy density
Source: BMC Plant Biol. 2023 Dec 11;23:636. doi: 10.1186/s12870-023-04635-8 (PMC10712098; doi:10.1186/s12870-023-04635-8)

Supplementary Table 1. Fatty acid profile (% total FA) and total fatty acids (% Leaf DW); (A) in DGAT+CO and null plants grown in the field where all plants are either infected (E+) or non-infected (E-) with AR1 fungal endophyte and (B) homozygous, hemizygous or null DGAT+CO plants grown in the field where all plants which are infected (E+) with AR37 fungal endophyte. Means (± SE). *n*=9 for (A) and *n*=21 for (B) field swards. Letters A, B, C indicate significant difference within each class of FA at P<0.05.

Supplementary Figure 1. Herbage yield of DGAT+CO T_2_ and nulls grown in field swards under regular defoliation with individuals either infected (E+) or non-infected (E-) with fungal endophyte AR1. Data points represent means ± SE, *n*=9.

Supplementary Figure 2. Binary vector pCAMBA1300 containing the coding sequences for cysteine Oleosin and DGAT (S205A) mutation, expressed from the rice CAB and RUBISCO small subunit promoters, respectively (see Winichayakul *et al*. 2008).

Supplementary Table 2. *In planta* alkaloid concentrations (µg/g) for each of the endophyte infected DGAT+CO treatments including (A) AR1 infected DGAT+CO grown in the growth chamber, (B) AR1 infected DGAT+CO grown in the field and (C) AR37 infected DGAT+CO grown in the field. Means (±SE).

Supplementary Figure 3. A diagrammatic representation of the crosses for developing T_2_ populations segregating for DGAT+CO which are either infected (E+) or not infected (E-) with *Epichloë*. Panel (A) shows crosses with DGAT+CO T_0_ event ODR4501 used to introduce strain AR1 and (B) shows crosses with DGAT+CO T_0_ event RCR5101 used to introduce strain AR37.

Supplementary Table S1.

1. AR1 Field Trial

| Harvest 3 | C16:0 | C16:1 | C18:0 | C18:1 | C18:2 | C18:3 | Total FA (%DW) |
| --- | --- | --- | --- | --- | --- | --- | --- |
| DGAT+CO/E+ | 19.31^A^ (0.46) | 2.17^B^ (0.07) | 2.23 (0.13) | 6.89^A^ (0.55) | 26.03^A^ (0.55) | 43.37^A^ (0.91) | 2.61^A^ (0.13) |
| DGAT+CO/E- | 19.84^A^ (0.31) | 1.82^C^ (0.09) | 2.28 (0.10) | 8.10^A^ (0.67) | 26.33^A^ (0.66) | 41.62^A^ (0.97) | 2.30A^B^ (0.11) |
| null/E+ | 21.57^B^ (0.14) | 2.37A^B^ (0.08) | 2.43 (0.12) | 3.83^B^ (0.34) | 20.34^B^ (0.77) | 49.46^B^ (1.21) | 2.10^B^ (0.05) |
| null/E- | 22.03^B^ (0.28) | 2.41^A^ (0.04) | 2.28 (0.04) | 3.98^B^ (0.15) | 19.38^B^ (0.45) | 49.92^B^ (0.61) | 2.02^B^ (0.06) |

| Harvest 4 | C16:0 | C16:1 | C18:0 | C18:1 | C18:2 | C18:3 | Total FA (%DW) |
| --- | --- | --- | --- | --- | --- | --- | --- |
| DGAT+CO/E+ | 16.06^A^ (0.53) | 1.26 (0.09) | 1.87 (0.16) | 7.32^A^ (0.83) | 26.33^A^ (1.17) | 47.16^A^ (2.14) | 3.35^A^ (0.17) |
| DGAT+CO/E- | 16.45^A^ (0.16) | 1.31 (0.05) | 1.77 (0.12) | 7.24^A^ (0.58) | 23.43^A^ (1.01) | 49.80^A^ (1.30) | 3.51^A^ (0.04) |
| null/E+ | 18.24^B^ (0.32) | 1.46 (0.10) | 1.71 (0.05) | 3.69^B^ (0.22) | 18.06^B^ (1.14) | 56.85^B^ (1.50) | 2.68^B^ (0.12) |
| null/E- | 18.44^B^ (0.16) | 1.35 (0.05) | 1.78 (0.11) | 4.12^B^ (0.26) | 19.05^B^ (1.16) | 55.25^B^ (1.36) | 2.84^B^ (0.06) |

| Harvest 5 | C16:0 | C16:1 | C18:0 | C18:1 | C18:2 | C18:3 | Total FA (%DW) |
| --- | --- | --- | --- | --- | --- | --- | --- |
| DGAT+CO/E+ | 14.10 (0.36) | 1.97^A^ (0.06) | 1.62 (0.08) | 7.62^A^ (0.48) | 22.34^A^ (0.39) | 52.35^A^ (0.70) | 3.86^A^ (0.12) |
| DGAT+CO/E- | 14.41 (0.29) | 1.89^A^ (0.09) | 1.80 (0.15) | 7.62^A^ (0.51) | 21.95^A^ (0.46) | 52.33^A^ (0.91) | 3.63^A^ (0.11) |
| null/E+ | 14.55 (0.34) | 2.33^B^ (0.03) | 2.08 (0.30) | 2.87^B^ (0.36) | 13.33^B^ (0.38) | 64.84^B^ (1.03) | 3.00^B^ (0.07) |
| null/E- | 14.92 (0.31) | 2.27^B^ (0.06) | 1.89 (0.18) | 2.61^B^ (0.16) | 13.31^B^ (0.35) | 65.00^B^ (0.78) | 3.10^B^ (0.06) |

1. AR37 Field Trial

| Harvest 4 | C16:0 | C16:1 | C18:0 | C18:1 | C18:2 | C18:3 | Total FA (%DW) |
| --- | --- | --- | --- | --- | --- | --- | --- |
| Hom  DGAT+CO/E+ | 16.17 (0.30) | 1.81 (0.07) | 2.07 (0.17) | 6.43 (0.70) | 25.21 (1.45) | 48.31 (2.03) | 2.94^A^ (0.07) |
| Hem  DGAT+CO/E+ | 17.14 (0.30) | 1.55 (0.11) | 2.29 (0.10) | 8.08 (0.71) | 27.46 (1.35) | 43.48 (1.99) | 2.68^B^ (0.03) |
| null/E+ | 16.58 (0.30) | 1.60 (0.11) | 1.98 (0.10) | 7.21 (0.77) | 27.34 (1.45) | 45.29 (2.01) | 2.12^C^ (0.02) |

| Harvest 5 | C16:0 | C16:1 | C18:0 | C18:1 | C18:2 | C18:3 | Total FA (%DW) |
| --- | --- | --- | --- | --- | --- | --- | --- |
| Hom  DGAT+CO/E+ | 12.87^A^ (0.20) | 1.71^A^ (0.04) | 1.78^A^ (0.02) | 10.73^A^ (0.08) | 28.57^A^ (0.15) | 44.34^A^ (0.23) | 3.91^A^ (0.06) |
| Hem  DGAT+CO/E+ | 13.81^B^ (0.12) | 1.80^B^ (0.03) | 1.80^A^ (0.02) | 6.98^B^ (0.20) | 23.55^B^ (0.34) | 52.06^B^ (0.47) | 3.29^B^ (0.04) |
| null/E+ | 16.14^C^ (0.28) | 2.14^C^ (0.02) | 1.40^B^ (0.03) | 2.29^C^ (0.09) | 13.49^C^ (0.24) | 64.53^C^ (0.42) | 2.46^C^ (0.04) |

Supplementary Figure S1.


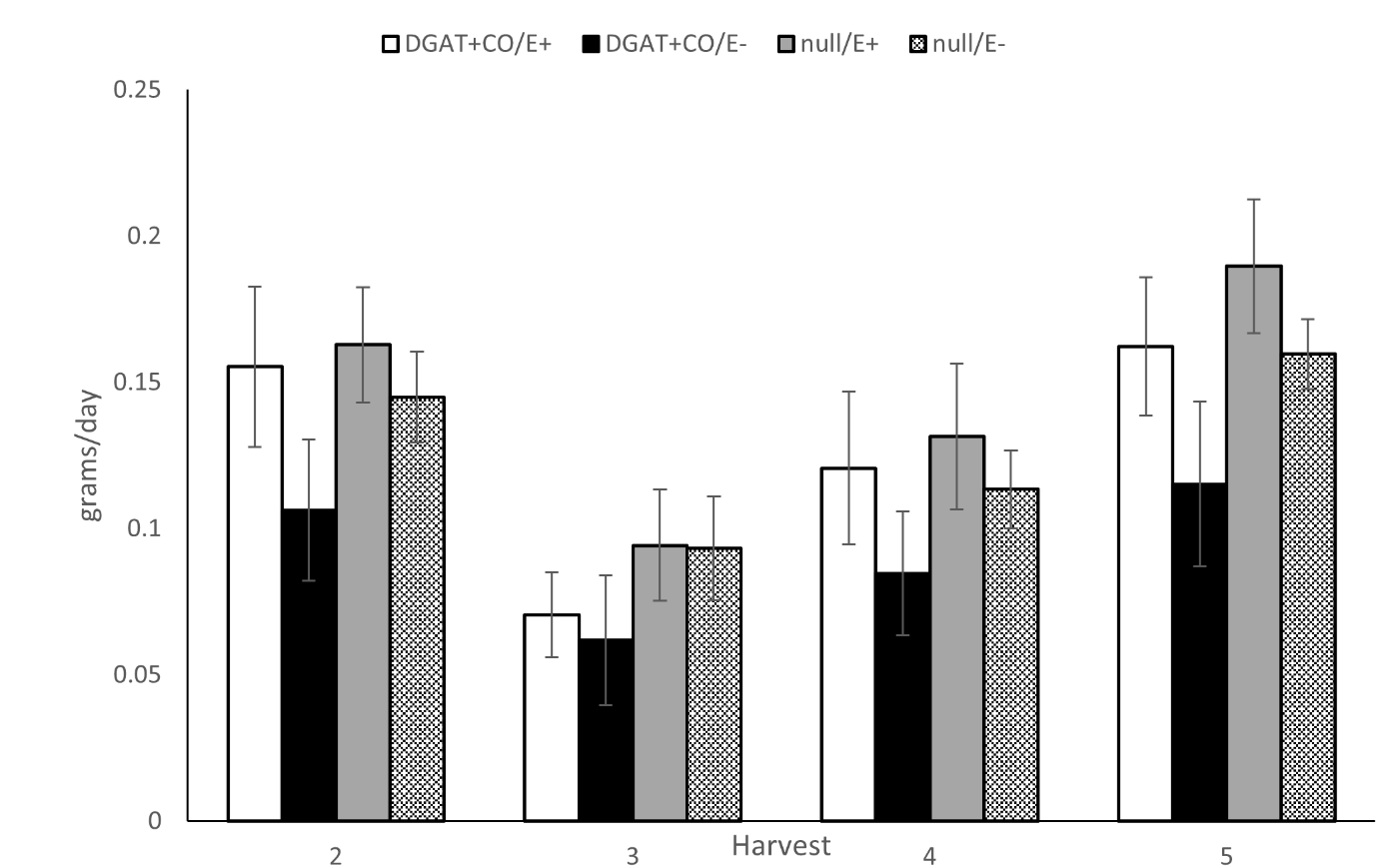


Supplementary Figure S2.


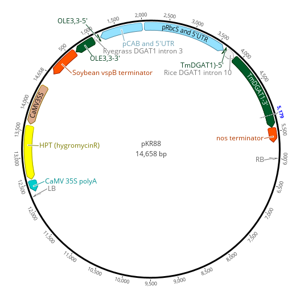


Supplementary Table S2.

1. AR1 Growth Chamber

| Growth Chamber | Pseudostem | | Leaf | |
| --- | --- | --- | --- | --- |
|  | DGAT+CO | null | DGAT+CO | null |
| Mycelial mass (mg/g) | 2.10 (0.36) | 2.07 (0.18) | 1.53 (0.26) | 1.75 (0.25) |
| Peramine (µg/g) | 68.73 (5.16) | 70.93 (5.38) | 58.18 (7.60) | 86.24 (6.50) |
| Paspaline | 3.30 (0.45) | 2.94 (0.43) | 0.39 (0.05) | 0.56 (0.07) |
| Terpendole E | 0.73 (0.09) | 0.66 (0.06) | 0.20 (0.03) | 0.26 (0.03) |
| Paspaline B | 0.15 (0.04) | 0.14 (0.04) | 0 | 0.01 (0.01) |
| 13-Desoxypaxilline | 0.90 (0.24) | 0.70 (0.19) | 0.01 | 0.01 |
| Paxilline | 0.49 (0.10) | 0.46 (0.10) | 0.01 | 0.02 (0.01) |
| Paxitriol | 0.14 (0.06) | 0.09 (0.02) | 0.01 | 0.01 |
| Terpendole I | 1.98 (0.65) | 2.06 (0.42) | 0.73 (0.23) | 0.62 (0.08) |
| Terpendole J | 0.03 (0.01) | 0.02 (0.01) | 0.01 | 0.01 |
| Terpendole C | 0.13 (0.06) | 0.06 (0.03) | 0.02 (0.01) | 0.01 (0.01) |
| Terpendole K | 0.48 (0.15) | 0.28 (0.16) | 0.01 | 0 |
| Terpendole M | 0.06 (0.03) | 0.04 (0.03) | 0.04 (0.04) | 0.02 (0.01) |
| Terpendole A | 0 | 0 | 0 | 0 |
| Terpendole N | 3.55 (0.83) | 3.05 (1.48) | 0.02 (0.01) | 0.03 (0.01) |

1. AR1 Field Trial

| Field Harvest 3 |  | |
| --- | --- | --- |
|  | DGAT+CO | null |
| Mycelial mass (mg/g) | 1.14 (0.12) | 1.25 (0.16) |
| Peramine (µg/g) | 13.97 (1.57) | 17.61 (0.97) |
| Paspaline | 1.04 (0.12) | 1.88 (0.11) |
| Terpendole E | 0.54 (0.07) | 1.32 (0.16) |
| Paspaline B | 0 | 0 |
| 13-Desoxypaxilline | 0 | 0 |
| Paxilline | 0 | 0 |
| Paxitriol | 0.08 (0.01) | 0.15 (0.01 |
| Terpendole I | 0.07 (0.02) | 0.19 (0.02) |
| Terpendole J | 0.09 (0.02) | 0.15 (0.02) |
| Terpendole C | 0.02 (0.01) | 0.05 (0.01) |
| Terpendole K | 0.04 (0.01) | 0.09 (0.02 |
| Terpendole M | 3.21 (0.35) | 5.51 (0.85 |
| Terpendole A | 0.09 (0.01) | 0.16 (0.01) |
| Terpendole N | 0.42 (0.06) | 1.03 (0.12) |

| Field Harvest 4 |  | |
| --- | --- | --- |
|  | DGAT+CO | null |
| Mycelial mass (mg/g) | 1.59 (0.23) | 1.76 (0.20) |
| Peramine (µg/g) | 13.0 (1.47) | 17.73 (1.45) |
| Paspaline | 1.17 (0.25) | 1.85 (0.31) |
| Terpendole E | 0.17 (0.04) | 0.34 (0.04) |
| Paspaline B | 0.01 | 0.02 (0.01) |
| 13-Desoxypaxilline | 0.04 (0.01) | 0.06 (0.01) |
| Paxilline | 0.01 (0.01) | 0.01 (0.01) |
| Paxitriol | 0.05 (0.01) | 0.10 (0.02) |
| Terpendole I | 0.06 (0.01) | 0.14 (0.02) |
| Terpendole J | 0.07 (0.01) | 0.05 (0.01) |
| Terpendole C | 0.08 (0.02) | 0.09 (0.01) |
| Terpendole K | 0.09 (0.02) | 0.09 (0.02) |
| Terpendole M | 2.82 (0.50) | 3.97 (0.61) |
| Terpendole A | 0.06 (0.01) | 0.05 (0.01) |
| Terpendole N | 0.41 (0.08) | 0.51 (0.10) |

| Field Harvest 5 |  | |
| --- | --- | --- |
|  | DGAT+CO | null |
| Mycelial mass (mg/g) | 1.00 (0.20) | 1.13 (0.09) |
| Peramine (µg/g) | 11.18 (0.91) | 16.18 (1.28) |
| Paspaline | 0.21 (0.31) | 0.37 (0.04) |
| Terpendole E | 0.06 (0.01) | 0.10 (0.01) |
| Paspaline B | 0 | 0 |
| 13-Desoxypaxilline | 0 | 0.01 |
| Paxilline | 0 | 0 |
| Paxitriol | 0.01 | 0.02 |
| Terpendole I | 0.06 (0.01) | 0.10 (0.01) |
| Terpendole J | 0.05 (0.01) | 0.04 (0.01) |
| Terpendole C | 0.03 (0.01) | 0.03 |
| Terpendole K | 0.05 (0.02) | 0.04 (0.01) |
| Terpendole M | 2.15 (0.43) | 2.44 (0.70) |
| Terpendole A | 0.06 (0.01) | 0.05 (0.01) |
| Terpendole N | 0.50 (0.12) | 0.49 (0.09) |

1. AR37 Field Trial

|  | Harvest 4 | | | Harvest 5 | | |
| --- | --- | --- | --- | --- | --- | --- |
|  | Homozygous | Hemizygous | null | Homozygous | Hemizygous | null |
| Mycelial mass (mg/g) | 2.77 (0.12) | 3.02 (0.20) | 3.19 (0.25) | 2.44 (0.15) | 2.90 (0.34) | 2.83 (0.21) |
| Total epoxyjanthitrems (µg/g) | 22.70 (1.83) | 33.04 (1.55) | 37.54 (3.03) | 19.47 (2.24) | 25.26 (2.48) | 26.64 (1.88) |
| E-Jantriol | 2.91 (0.20) | 4.23 (0.29) | 4.90 (0.43) | 2.77 (0.26) | 3.36 (0.40) | 3.75 (0.26) |
| E-Jan I | 8.51 (0.72) | 12.78 (0.62) | 15.50 (1.43) | 6.62 (0.86) | 8.94 (0.86) | 10.37 (0.77) |
| E-Jan II | 4.94 (0.38) | 6.78 (0.28) | 7.08 (0.44) | 4.25 (0.43) | 5.52 (0.57) | 5.16 (0.36) |
| E-Jan III | 5.02 (0.42) | 7.31 (0.35) | 7.95 (0.58) | 4.27 (0.48) | 5.37 (0.54) | 5.25 (0.38) |
| E-Jan IV | 1.33 (0.14) | 1.94 (0.07) | 2.11 (0.18) | 1.56 (0.22) | 2.06 (0.16) | 2.12 (0.15) |

Supplementary Figure S3.


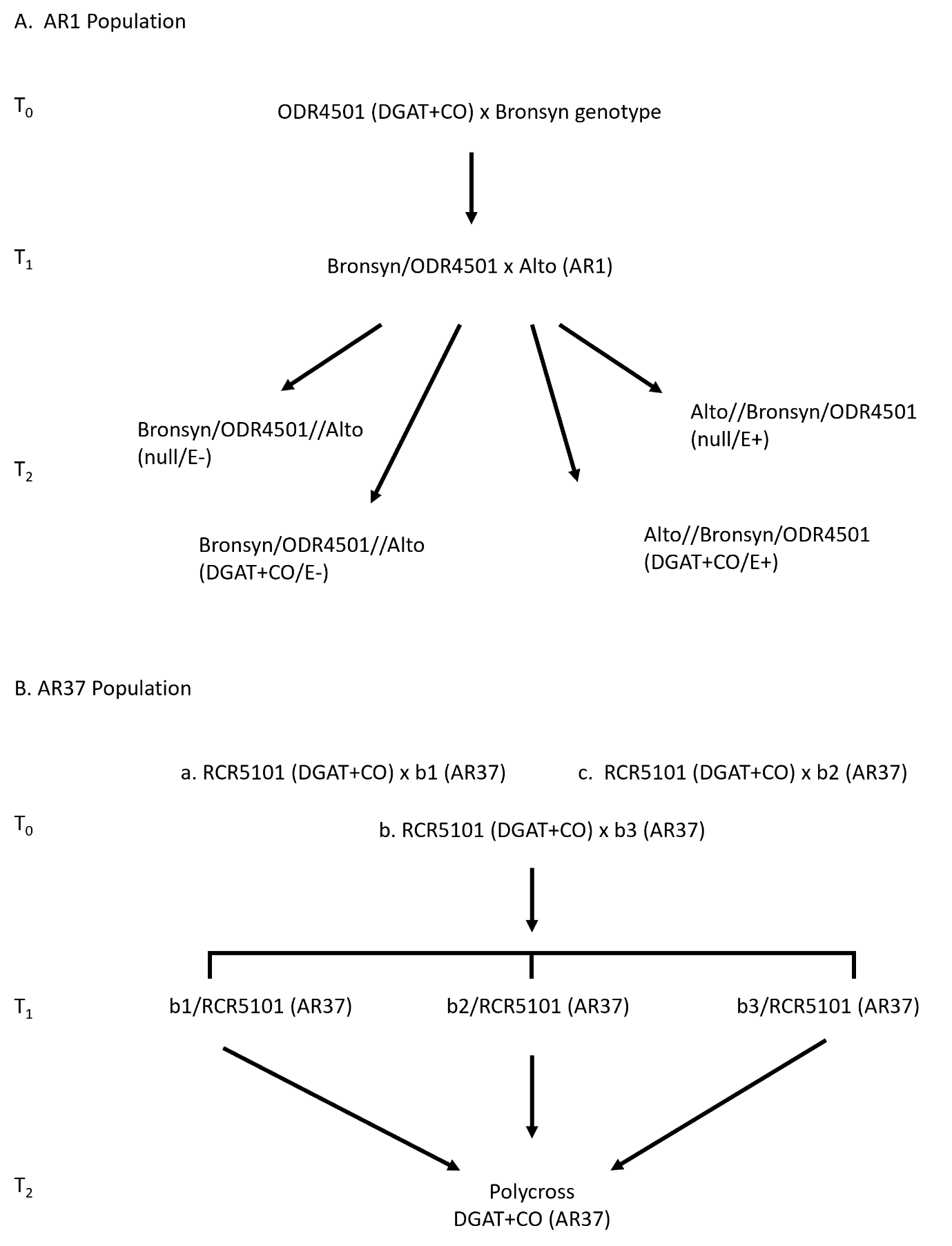

Supplement: Supplementary file 1 — Additional file 1: Supplementary Table S1. Fatty acid profiles in DGAT + CO and null plants grown in the field. Supplementary Figure S1. Herbage yield of DGAT + CO T2 and nulls grown in field. Supplementary Figure S2. Binary vector map of the DGAT + CO expression construct. Supplementary Table S2. Full in planta alkaloid analysis of endophyte infected DGAT + CO. Supplementary Figure S3. Diagrammatic representation of the crosses for developing T2 populations. [file 12870_2023_4635_MOESM1_ESM.docx]
